# Extirpation of a Tubo-Ovarian Cys

**Published:** 1893-10

**Authors:** R. A. Archibald


### EXTIRPATION OF A TUBO-OVARIAN CYST.

By Dr. R. A. Archibald.*

A short time ago, at his hospital on Broadway, Dr. Pierce, of Oakland, California, performed the above operation with success. The doctor was ably assisted by Dr. H. A. Spencer, and Dr. Rowland Lord, both of San Jose.

The subject was a six-year-old thoroughbred mare called Lady Emily, sired by Three Cheers, dam Queen Emma by Woodburn, bred by John Arnet, of Pleasanton, Cal., and at present owned by J. B. Chase, of San Francisco, and handled by the well known trainer, Thos. G. Jones.

The mare, since she was three years old, has been bred to several first class horses, such as Imp. Cheviot, Imp. Friar Tuck, etc., but without success. Last June Mr. Jones, seeing that she was rapidly losing her health, and in fact she commenced declining so fast that it became absolutely necessary to have some medical or surgical attention for her, had her examined by several reputable veterinarians, who diagnosed her case as prolapsus uteri, and she was considered by them as incurable. The mare kept getting worse, until Mr. Jones decided to take her to Oakland, and have her examined by Dr. Pierce, who (as will be shown later) correctly diagnosed her trouble as being due to a disease of the right ovary, and decided that it would be necessary to extirpate the diseased organ before it would be possible for the mare to regain her normal condition.

He found that there was a continual discharge of a reddish-brown fluid mixed with mucus from the genital organs, and the uterus and vagina were in a highly catarrhal condition. The right

*Read before the California State Veterinary Medical Association.

fallopian tube was also found to be enlarged and indurated to the touch.

Having gained permission from the owner to operate on the mare, he invited Drs. Spencer and Lord to assist him.

He then proceeded to prepare the animal as follows: a few days prior to the operation laxatives were administered until the fluid, slightly fæculent evacuations, showed that the intestinal canal had been emptied. The uterus and vagina were washed out twice daily with a weak solution of phynele. The animal was pronounced ready for the operation on July 31st, when the visiting surgeons, on Dr. Pierce's invitation, made a thorough examination, agreeing with Dr. Pierce in that the removal of the right ovary was necessary to save the animal's life. Owing however to the inflammatory and catarrhal condition of the vagina, it was decided not to operate in the usual manner through the vagina, but through the flank. Two hours before the operation the rectum was emptied by an enema. Only a small number of instruments were selected and placed in readiness in an antiseptic solution of phynele, such as bistouries, artery forceps, scissors, suture material, a couple of sponges, a pacqalin cautery for cauterizing the bleeding surfaces.

The subject was placed on the operating table and put under the influence of chloroform. The abdominal wall was well washed with soap and water and an antiseptic solution The hairs and upper layers of the epidermis were removed with the razor off a place midway between the last rib, the transverse processes of the lumbar vertebræ and the anterior iliac spine. A spray was suspended over the field of operation, the spray being filled with a solution of lysol, the parts operated on were by this method kept continually saturated. An incision was then made through the skin and *panniculus carnosus;* the fibers of the abdominal muscles were separated by the finger until an opening was made large enough to admit the hand of the operator; the hand was then inserted through the peritoneum into the abdominal cavity; the ovary was grasped and brought to the opening where, with the scalpel, the peritoneum was severed and the ovary, with a portion of the fallopian tube, was removed with the ecraseur. Peritoneal toilette was dispensed with, as very little blood had escaped into the abdominal cavity. The peritoneum was then sutured with carbolized catgut, the ends of the sutures being left long enough to act as a drainage tube; the muscles and skin were sutured with silk. A dressing, composed of Hyd. Chl. Mite two drachms and

Acid Boricum one ounce, was dusted on the wound; three yards of antiseptic gauze (mussed up) was put over this, and over this was placed about a quarter of a pound of absorbent cotton, and over all a strong muslin roller, wound round the abdomen and secured so it was impossible for it to slip forward or backward. The after treatment consisted of two-drachm doses of Ex. Arnicæ Fl. every hour for the first twenty-four hours, and three times daily afterwards. A tepid solution of sodium chloride and zinci chloridum was injected into the uterus twice daily.

Two hours after the operation it was found necessary to administer two grains Morphiæ Sulph. hypodermically, in order to quiet the animal, as she was very nervous.

The dressing was removed at the end of two weeks, when it was found that the wound had nearly healed, and there was no trace of pus having formed under the dressing. A fresh dressing similar to the first was applied and left there until the wound had perfectly healed.

On the morning after the operation her temperature was 102°, pulse 48, and from that time on the pulse and temperature gradually decreased until they reached their normal condition.

The mare at the present time is healthier and more thrifty than she has been for four years, and it is thought by the attending veterinarians that she may become pregnant next year.

Upon examining the extirpated tumor, it was found to be what is commonly called a "Tubo-Ovarian Cyst." The outer surface of the ovary was perfectly smooth. The cyst was as large as two fists and contained a considerable quantity of thick brownish red fluid. The inner surface presented warty excrescences in places. The fimbriated portion of the tube took part in the formation of the cyst, so the index finger could be passed from the cyst into the tube.

As was mentioned before, the extirpated tumor (which looked somewhat like an ordinary cystoma) also contained a portion of the fallopian tube, which was dilated and thickened. The medium portion of the fallopian tube presented a broad mesosalpinx, while the lateral portion ran into the wall of the tumor. On being opened the tube discharged a thick brownish red fluid, and could be followed laterally into the cyst. When seen from the cyst, the opening of the tube was markedly convoluted, and the fimbria passed over upon the inner surface of the cyst, as prolongations of the longitudinal folds. It would be well to say here, that the entire tumor was covered with peritoneum, and but a comparatively small portion was situated in the broad ligament. In regard to the adhesion of the tube to the ovary (since it is not a part of physiological ovulation), it must have preceded the rupture of the cyst, and the formation of tubo-ovarian cysts is due to the assumption of a catarrh of the tube and follicle.

The tubal catarrh causes circumscribed peritonitis and adhesion of the tube. The catarrh of a follicle causes its dilatation, and its rupture into the tube produces the tubo-ovarian cyst. The first stage in the process is adhesion of the fimbriæ, which can occur only on the peritoneal surface. The fimbriæ and their terminations are therefore directed inwards. This results in dilatation and dropsy of the tube. If a follicle now matures or forms a serous cyst and ruptures into the tube, the tubo-ovarian cyst is formed. Further secretion of the tube will enlarge the ovarian part of the cyst. This explains the fact that the ends of the fimbriæ are found upon the inner surface of the cyst. The reason that this cyst did not increase in size is due to the fact that the tube was always pervious in the direction toward the uterus, and increased pressure within the cyst occasionally gave rise to a discharge of fluid into the uterus. Thanking you for your kind attention, and renewing the hope that in the near future the fruits derived from the discussion which I sincerely hope this will provoke, may be beneficial to all of us.

